# Methionine restriction on oxidative stress and immune response in dss-induced colitis mice

**DOI:** 10.18632/oncotarget.17812

**Published:** 2017-05-11

**Authors:** Gang Liu, Lei Yu, Jun Fang, Chien-An Andy Hu, Jie Yin, Hengjia Ni, Wenkai Ren, Veeramuthu Duraipandiyan, Shuai Chen, Naif Abdullah Al-Dhabi, Yulong Yin

**Affiliations:** ^1^ Key Laboratory of Agro-Ecological Processes in Subtropical Region, Institute of Subtropical Agriculture, Chinese Academy of Sciences, Changsha 410125, China; ^2^ China Animal Disease Control Center, Beijing 102618, China; ^3^ College of Bioscience and Biotechnology, Hunan Agricultural University, Changsha 410128, China; ^4^ Department of Biochemistry and Molecular Biology, University of New Mexico School of Medicine, Albuquerque, New Mexico 87131, USA; ^5^ Addiriyah Research Chair for Environmental Studies, Department of Botany and Microbiology, College of Science, King Saud University, Riyadh 11451, Saudi Arabia; ^6^ College of Animal Science, South China Agricultural University, Guangzhou 510642, China; ^7^ Laboratory of Animal Nutrition and Human Health, School of Life Sciences, Hunan Normal University, Changsha 410081, China

**Keywords:** inflammatory bowel disease, methionine restriction, diet, oxidative stress, NF-κB

## Abstract

A strong correlation exists between inflammatory bowel disease (IBD) and oxidative stress involving alterations of several key signaling pathways. It is known that methionine promotes reactive oxygen species (ROS) production; we therefore hypothesize that a methionine restriction diet would reduce ROS production, inflammatory responses, and the course of IBD. We generated a murine colitis model by dextran sodium sulfate (DSS) treatment and tested the effects of the methionine restriction diet. Forty-eight mice were randomly divided into four groups of equal size, which included a control (CON) group, an MR (methionine restriction diet) group, a DSS treated group and an MR-DSS treated group. Mice in the first two groups had unrestricted access to water for one week. Mice in the two DSS-treated groups had unrestricted access to 5% DSS solution supplied in the drinking water for the same period. Mice in the CON and DSS groups were given a basal diet, whereas mice in the MR-DSS and MR groups were fed a 0.14% MR diet. We found that DSS reduced daily weight gain, suppressed antioxidant enzyme expression, increased histopathology scores and activated NF-κB and nuclear factor erythroid 2-related factor 2/Kelch-like ECH-associated protein 1 (Nrf2/Keap1) signaling. We also showed that the MR diet upregulated catalase (CAT), superoxide dismutase (SOD), and glutathione peroxidase (GPx) activities, decreased myeloperoxidase (MPO), TNF-α and IL-1β, and reversed activation of the NF-κB signaling pathway in MR-DSS mice. Taken together, our results imply that the MR diet may be considered as an adjuvant in IBD therapeutics.

## INTRODUCTION

Inflammatory bowel disease (IBD) is a chronic disorder of the gastrointestinal tract with increasing incidence around the world [1]. IBD, including ulcerative colitis (UC) and Crohn's disease, causes inflammation primarily affecting the gut mucosa and submucosa and is believed to be the result of a dysregulated immune response associated with environmental and genetic factors [2]. These factors include the microbiota, oxidative stress and diet [3, 4]. As part of the inflammatory process, the lamina propria of the gut is infiltrated by significant numbers of immune cells, which include eosinophils, polymorphonuclear neutrophils (PMNs) and activated macrophages. Once the lamina propria is infiltrated, the abovementioned cells generate a large quantity of reactive oxygen species (ROS) [5]. As ROS can cause lipid, protein and DNA oxidation and damage, it is believed that oxidative stress plays a critical role in the development of and/or the exacerbation of the symptoms of numerous diseases, including IBD [3]. In addition, the DNA transcription factor, nuclear factor-κB (NF-κB) and other gene expression regulators are crucially involved in the expression of pro-inflammatory cytokines, which are important for IBD initiation and pathogenesis [6]. An increasing use of immunomodulator and biologic therapies in the treatment of IBD has been reported.

Adverse effects and safety concerns limit the use of many pharmaceutical options (for example, antibody therapy, immune-modulators and steroids) for the treatment of IBD. Malignancy and infection are two of the major side effects [7, 8]. The past two decades have witnessed the development of a number of experimental models designed to understand the pathophysiology and nutritional and pharmacological interventions of IBD in humans [9]. The major reason is that there are similarities in etiology, pathogenesis and therapeutic response between animal models and humans with IBD (particularly in UC). One of the commonly used methodologies is dextran sodium sulfate (DSS)-induced acute colitis [10], in which animals present an increased leukocyte recruitment response and production of cytokines and other inflammatory mediators, leading to tissue damage [11]. In addition, the expression of a number of genes (such as pro-inflammatory cytokines) important in the immune response, inflammation and oxidative damage are influenced by DSS treatment [12].

Interestingly, the use of methionine restriction (MR) diet increases the lifespan of a wide range of organisms, including yeast [13], fruit fly [14] and rodents [15], and decreases the onset, progression and incidence of several neurodegenerative conditions [16]. Furthermore, decreased methionine ingestion increases mitochondrial biogenesis [13] and reduces mitochondrial oxidative damage [17]. Moreover, methionine exhibits a regulatory effect on the electron transport chain of mitochondria, and when methionine is restricted from the diet, the amounts of mitochondrial complexes and ROS in rodents are significantly reduced [18]. However, whether an MR diet has any beneficial effect on IBD has not been investigated.

Previously, we reported the role of amino acids in nutritional regulation of the inflamed gut in animal models and demonstrated that some amino acids can be used as modulators of intestinal inflammatory diseases [19]. In this study, the impact of an MR diet on the immune response and oxidative stress was exploited in a mouse model of colitis.

## RESULTS

**Figure 1:**
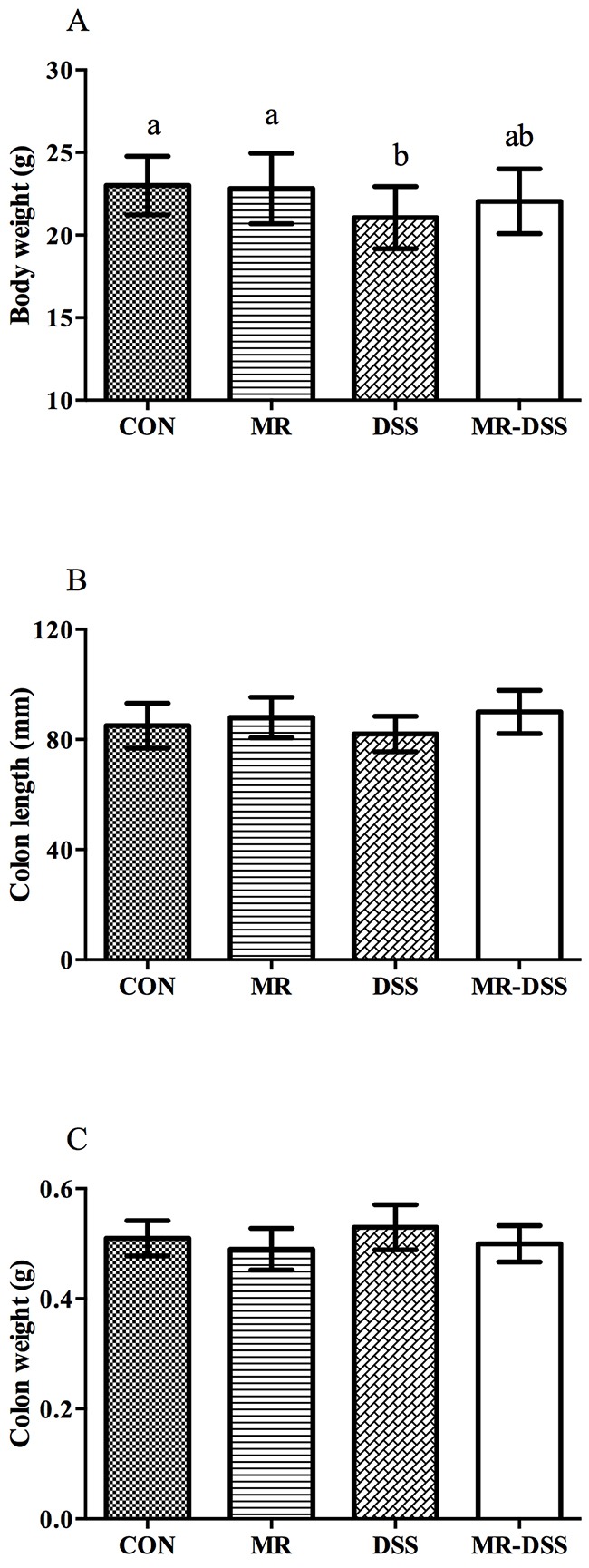
The effect of administration of an MR diet after DSS treatment on (A) body weight, (B) colon length and (C) colon weight Data are presented as the mean ± SEM. Values with different superscript letters were significantly different (*P* < 0.05; *n* = 12).

**Figure 2:**
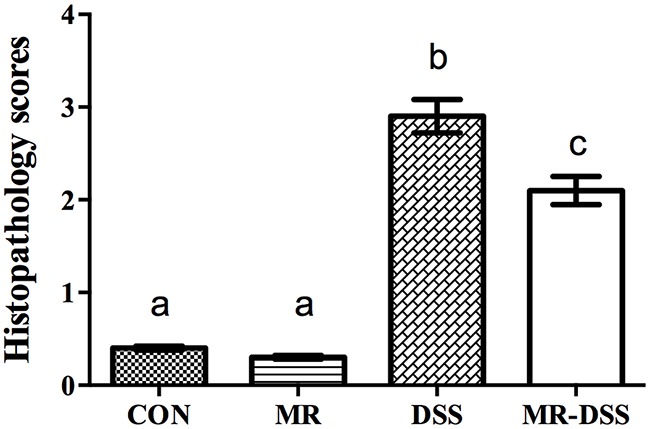
Histopathology scores given for mice in the control group and COS groups after 7 days of DSS administration Data are presented as the mean ± SEM. Values with different superscript letters were significantly different (*P* < 0.05; *n* = 12).

As the most frequently used index in the clinic for IBD is weight loss, we monitored the mean daily weight gain following DSS treatment, and found that a significant decrease (*P*< 0.05) in the body weight was observed in the DSS group (Figure 1A). A general tendency to reduce this suppression in growth was seen in MR-IBD mice (*P* > 0.05) (Figure 1A). There was no discernible difference in colon length or colon weight between the four groups (Figure 1B and 1C). Significantly more severe colitis was observed in the colons from DSS-treated mice compared to the MR-DSS mice following the administration of DSS (*P*< 0.05) (Figure 2).

The extent of neutrophil infiltration in the mouse colonic tissue was quantified by measuring the activity of myeloperoxidase (MPO), as this enzyme is most abundant in the granulocytes of neutrophils. The mucosa was infiltrated by inflammatory cells, particularly neutrophils, in addition to the intestinal inflammation induced by the DSS. Table 1 shows the MPO and antioxidant enzyme activities. The MR diet had no impact on the MPO activity compared to the control group. A significantly (*P* < 0.05) increased MPO activity in the DSS-induced colitis was observed compared to the mice in the CON and MR group; this increase was suppressed by the administration of the MR diet (*P*< 0.05).

**Table 1:** Colonic myeloperoxidase and antioxidant enzyme activities in mice following the induction of colitis by DSS administration

	CON (U/mg proteins)	DSS (U/mg proteins)	MR (U/mg proteins)	MR-DSS (U/mg proteins)
MPO	1.89±0.16 ^a^	2.42±0.21^b^	1.85±0.15^a^	2.13±0.24^c^
SOD	28.4±2.8^a^	20.2±1.8^b^	29.7±32.6^a^	22.5±1.9^c^
CAT	59.4±4.6^a^	48.3±3.7^b^	60.5±4.9^a^	53.4±3.9^c^
GPx	1.39±0.11^a^	1.05±0.09^b^	1.41±0.12^a^	1.19±0.11^c^
GRed	0.094±0.004	0.097±0.003	0.095±0.004	1.004±0.003

Data are presented as the mean ± SEM. Values with different superscript letters were significantly different.

DSS administration resulted in a decrease in CAT activity, SOD activity, and GPx activity (*P* < 0.05), compared to the control and MR groups, but no significant changes in SOD, CAT, GRed or GPx were observed between the control and MR groups. Comparing the MR-DSS group with the DSS group, the former resulted in significantly higher SOD, CAT, and GPx activities (*P*< 0.05).

**Figure 3:**
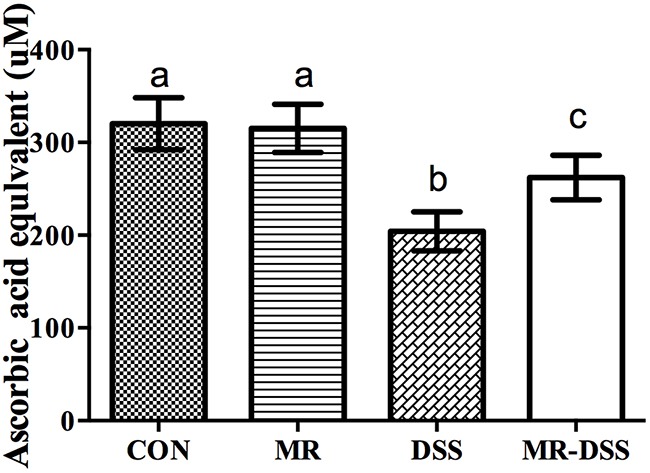
The effect of an MR diet on plasma antioxidant power The total antioxidant capacity was measured in the mouse plasma treated as per the protocol using the ABTS assay. Data are presented as the mean ± SEM. Values with different superscript letters were significantly different (*P* < 0.05; *n* = 8).

The 2,2′-azino-bis(3-ethylbenz-thiazoline-6-sulfonic acid) (ABTS) array was used to determine the antioxidant capacity in plasma. Figure 3 shows the impact of the administration of an MR diet on the plasma antioxidant power. There was a decrease in the DSS and MR-DSS groups compared with the MR and control groups (*P*< 0.05). There was a significantly increased total antioxidant capacity in the MR-DSS group compared to the DSS group (*P*< 0.05), but no significant difference between the MR group compared to the CON group.

**Figure 4:**
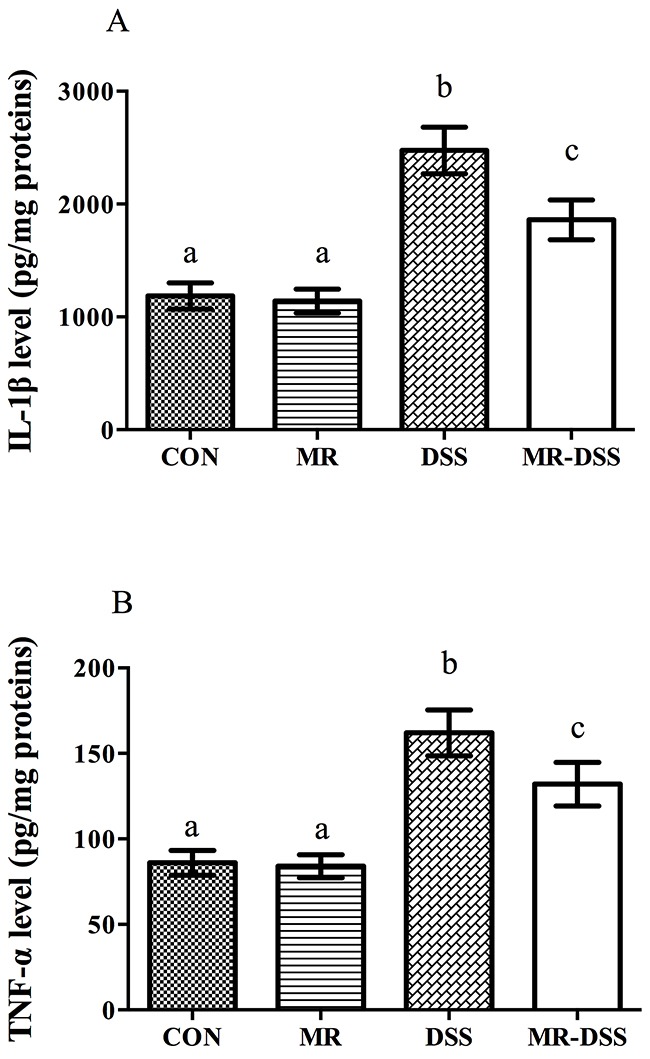
Colonic IL-1β (A) and TNF-α (B) levels of four groups Data are presented as the mean ± SEM. Values with different superscript letters were significantly different (*P* < 0.05; *n* = 8).

Figure 4 shows the colonic IL-1β and TNF-α levels of the four groups. The administration of the MR diet had no effects on the colonic level of the two compared to the CON group. However, there were significantly higher levels of both cytokines in the DSS and MR-DSS groups compared to both the CON and MR groups (*P* < 0.05). Both IL-1β and TNF-α levels were lower in the MR-DSS group that in the CON-DSS group (*P* < 0.05).

**Figure 5:**
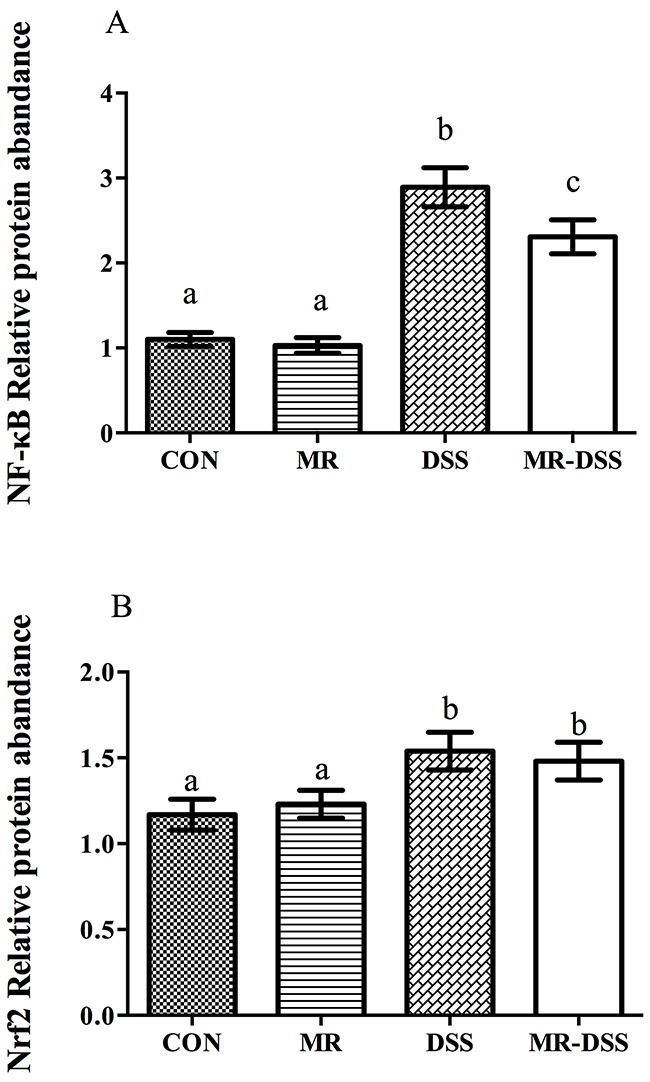
NF-κB (A) and Nrf2/Keap1 (B) signaling pathways in the DSS-induced colitis model Data are presented as the mean ± SEM. Values with different superscript letters were significantly different (*P* < 0.05; *n* = 8).

Measurements of nuclear NF-κB (p65) were performed to evaluate NF-κB activation in the colons of the mice. Treatment with DSS resulted in a significantly activated nuclear NF-κB signal (*P* < 0.05), as shown in Figure 5A. No significant difference in nuclear NF-κB (p65) was seen between MR mice and the CON group. In the MR-DSS group, the activation was significantly suppressed by the MR diet although there was a significantly lower quantity of nuclear NF-κB in the MR mice than in the MR-DSS group (*P* < 0.05). For the Nrf2/Keap1 signaling pathway, the MR diet had no impact on the Nrf2 signal compared to the control group as shown in Figure 5B, but there was clear enhancement of the colonic nuclear Nrf2 abundance resulting from DSS and MR-DSS groups compared to the control and MR groups (*P* < 0.05).

## DISCUSSION

IBD in humans is associated with intermittent periods of acute gastrointestinal tract inflammation [20, 21]. To investigate if an MR diet can reduce the symptoms of IBD, we selected a mouse colitis model that is induced by DSS. We found that 5% DSS induced acute colitis and reduced body weight in mice, which is consistent with previous work [10, 22]. Evidence has shown the presence of high levels of MPO in IBD [23], and DSS increases colonic MPO activity through the induction of neutrophil infiltration, which is a marker of the inflammatory response within the colonic mucosa [24]. We observed a significant increase of MPO activity in the mice of the DSS and MR-DSS groups. As the migration of neutrophils and the increase of MPO activity are the main characteristics of chronic inflammation of the colon, our results validate the importance of an MR diet. In addition, inflammation is associated with the recruitment and activation of phagocytic leukocytes within the mucosa, which produce and release a large quantity of superoxide anion and other ROS [5] in an uncontrolled manner, potentially causing cellular damage by overwhelming protective mechanisms [25]. Highly localized concentrations of ROS can attack and inactivate SOD and other antioxidant enzymes, preventing the neutralization of ROS. Thus, the significant reduction of SOD and CAT by DSS treatment contributes to the tissue damage, similar to the colitis phenotype in human IBD patients [26]. In fact, oxidative stress has two major effects: direct intestinal cell damage and initiation of inflammation through the activation of a number of signaling pathways, including NF-κB [5]. This class of complex proteins is known [3] to be associated with the expression of a range of chemokines, adhesion factors and cytokines in response to stress (for example, oxidative stress and inflammation). The transcription of many pro-inflammatory genes, including those of adhesion molecules, chemokines, iNOS and inflammatory cytokines, can also be activated by NF-κB. In addition, our DSS-treated mice also showed an increase of excessive NF-κB, but when administered an MR diet, a significant suppression of NF-κB activation, which is consistent with those in humans patients with IBD [26, 27].

Beloqui *et al*. reported that pro-inflammatory cytokines are actively secreted in inflamed regions in IBD, indicating that pro-inflammatory cytokines are directly and heavily involved in immunopathogenesis [28]. Mice treated with DSS exhibited a significant upregulation of IL-1β expression compared to the healthy control group, demonstrating that colonic inflammation was induced by 5% DSS. Our results showed that mice treated with DSS, regardless of whether the MR diet was administered, exhibited upregulated levels of IL-1β and TNF-α in colon samples, which is consistent with patients with IBD [29] and other animal models of IBD. TNF-α, a pleiotropic cytokine, is involved in numerous inflammatory processes relevant to IBD pathogenesis, including leukocyte recruitment, apoptosis, cytotoxic and phase response, intestinal permeability, and expression of adhesion molecules [30]. Thus, our results indicate that MR can be used as a potent immunomodulatory and anti-inflammatory modulator, and the MR diet should be considered as an adjuvant diet for animal models and human patients with IBD.

## MATERIALS AND METHODS

### Animal and experimental design

The animal work was performed in accordance with the Chinese Guidelines For Animal Welfare And Experimental Protocols and was approved by the Animal Care and Use Committee of the Institute of Subtropical Agriculture at the Chinese Academy of Sciences [31]. Forty male ICR mice, weighing approximately 21 g, were kept in cages constructed of polycarbonate material with access to laboratory strip chow at all times. The temperature within the cages was controlled at 25°C ± 3°C, and the humidity was kept at 50 ±5%. A 12-hour cycle of light and dark was used. The mice were randomly divided into four groups of equal size randomly: a control group (CON), a group treated with DSS only (DSS), a group treated with MR (MR), and a group treated with MR and DSS (MR-DSS). The CON and MR groups were given free access to water for a period of seven days. Mice in the CON and DSS groups were given a basal diet (methionine 0.8%) [19], and mice in the MR-DSS and MR groups were fed a 0.14% methionine MR diet. Each mouse in the DSS and MR-DSS groups were allowed seven days of free access to a 5% (wt/vol) DSS solution (molecular weight of 5,000 Dalton DSS (Kayon Biological Technology Co., Ltd.) dissolved in water), which was supplied to them as drinking water [19].

The morning of the eighth day, the average daily weight gain was calculated. Each mouse was then sacrificed, and the length and weight of the colon were measured and the colon tissue of each mouse was harvested and immediately frozen in liquid nitrogen for further analysis according to our published protocol [19].

### Histological analysis

Colon tissue was fixed in 10% formalin. Hematoxylin and eosin staining of paraffin-embedded sections was performed. Six criteria were used to grade colitis histologically, including inflammation, edema, erosion, cryptitis, ulcers and goblet cell hyperplasia [32]. Lesions were scored using a scale of zero to four. Zero represented no colitis or epithelial thickening, one represented an increased number of mucosal leukocytes and/or slight epithelial cell hyperplasia, two represented multiple loci of inflammation, leukocytic mucosal and submucosal infiltration and/or marked epithelial cell hyperplasia (defined as a twofold to threefold increase in crypts), three represented extensive mucosal and submucosal leukocytes, depletion of mucin-secreting goblet cells and/or pronounced epithelial cell hyperplasia (defined as a threefold to tenfold increase in crypts), and four represented extensive transmural leukocytic infiltration, crypt abscesses and/or a marked epithelial cell hyperplasia (defined as a greater than tenfold increase in crypts).

### Determination of colonic antioxidant enzyme activities

Protein concentrations were measured by a BCA assay kit (Pierce, Jiancheng Bioengineering Institute, Nanjing, China). Guaiacol oxidation by MPO-produced hydroxyl radicals in the presence of hydrogen peroxide was evaluated as previously described [33]. This activity, expressed in units per mg of protein, was defined as the quantity of enzyme degrading 1 μmol of hydrogen peroxide per minute. The colon sample sections were mechanically homogenized on ice in a 100 mM Trizma buffer pH 7.4 with 1 mM EDTA and 1 mM phenylmethanesulfonyl fluoride (PMSF). The homogenate was sonicated on ice for thirty seconds and subsequently centrifuged at 1,000 x g for ten minutes at 4°C. The supernatants were then aliquoted and stored at -80 °C until subsequent analysis. Antioxidant enzyme activities, defined as the quantity degrading 1 μmol per minute of substrate (in units per mg proteins), were ascertained in triplicate using catalase (CAT), superoxide dismutase (SOD), glutathione reductase (GRed) and glutathione peroxidase (GPx) kits (Jiancheng Bioengineering Institute, Nanjing, China).

### Plasma antioxidant power

A mixture containing 10 mM ABTS (2,2′-azino-bis(3-ethylbenz-thiazoline-6-sulfonic acid)) solution and 2 mM hydrogen peroxide was stored at 4°C in the dark overnight. The solution was diluted to a 1:10 ratio such that an absorbance on the order of 0.31 at 660 nm was obtained. One milliliter (ml) of buffer of pH 3.6, 10 μl of plasma and 25 μl of ABTS were added to a 10 ml cuvette. ABTS radical cation decolorization was used to determine the total antioxidant capacity of the plasma (one of the antioxidants is ascorbic acid). The absorbance was measured five minutes after at 660 nm. For each assay, a blank control was performed and each measurement was performed in triplicate. The results were presented as the micromolar equivalent of ascorbic acid [34].

### Cytokine measurement

Fifty micrograms of each colon tissue sample was lysed in a cell lysis kit (Jiancheng Bioengineering Institute, Nanjing, China). In brief, the samples were first rinsed in the wash buffer and then thawed in the lysis buffer, which contained PMSF (500 mM) and a protease inhibitor cocktail. The homogenates were further treated by a freeze-thaw cycle and sonicated on ice before being centrifuged at 4,500 x g for four minutes. Levels of IL-1β and TNF-α in the supernatants were assayed by a specific sandwich ELISA kit (ELISA Ready-SET-GO, eBioscience, CA, USA). The amount of cytokines was normalized by the protein level of the colonic samples.

### Nuclear protein extraction and western blot analysis

The extraction of nuclear and cytoplasmic proteins was performed according to the instructions of the manufacturer (Thermo Fisher Scientific, USA). For western blot analysis,each 50 μg sample was separated using SDS-polyacrylamide gel electrophoresis and transferred electrophoretically to a polyvinylidene difluoride (PVDF) membrane (Bio-Rad, Hercules, CA, USA). The membrane was first blocked using evaporated milk at a 7% concentration and diluted at room temperature in Tris-buffered saline containing 0.1% Tween 20 for no less than two hours, and then incubated at 4°C with either NF-κB (p65) or Nrf2 primary antibodies overnight. For the loading control, we used antibodies against PCNA (Sigma) and β-actin (Sigma) as the primary antibodies for the nuclear and cytoplasmic samples, respectively, and alkaline phosphatase-conjugated anti-rabbit or anti-mouse IgG antibodies (Promega, Madison, WI, USA) as secondary antibodies for the corresponding primary antibodies. The signals were quantified and then analyzed digitally using ImageJ (NIH), subtracting the measured intensity of each band from the background intensity. The target proteins’ expression ratio was normalized against PCNA or β-actin.

### Statistical analysis

We performed general statistical analysis using SPSS version 22.0, and one-way ANOVA in group comparisons to determine the homogeneity of Levene's test and Tukey's multiple comparison test. The data are expressed as the mean ± standard error of the mean. Identical superscripts assigned to any values in a given row indicate that those values are not significantly differently at the *P*> 0.05 level; different superscripts indicate significant differences between the values in a given row at the same level (*P* < 0.05).

## Abbreviations

IBD: inflammatory bowel disease
MR: methionine restriction
DSS: dextran sodium sulphate
CAT: catalase
SOD: superoxide dismutase
MPO: myeloperoxidase
GPx: glutathione peroxidase
UC: ulcerative colitis
ROS: reactive oxygen species
ABTS: 2,2′-azino-bis(3-ethylbenz-thiazoline-6-sulfonic acid)
